# Depression as a Longitudinal Modulator of Physical–Verbal Aggression in Early Adolescence: Trajectories and Sex Comparisons

**DOI:** 10.62641/aep.v54i4.2265

**Published:** 2026-08-15

**Authors:** Rodolfo Gordillo, Victoria del Barrio, Miguel Ángel Carrasco

**Affiliations:** ^1^Department of Psychology and Criminology, Open University of Madrid (UDIMA), 28400 Collado Villalba, Spain; ^2^Department of Personality, Evaluation and Psychological Treatments, National University of Distance Education (UNED), 28040 Madrid, Spain

**Keywords:** depression, physical–verbal aggression, early adolescence, longitudinal study, sex differences, comorbidity

## Abstract

**Background:**

Depression during early adolescence has been associated with increased aggressive behaviour; however, longitudinal evidence focused on this developmental stage remains limited, and findings regarding sex differences are inconsistent. In addition, few studies have examined physical and verbal aggression jointly across early adolescence. This study aims to examine the longitudinal association between depression and physical–verbal aggression during early adolescence and to analyse whether sex moderates the developmental trajectories and severity of aggression.

**Methods:**

A three wave longitudinal study was conducted with 251 adolescents (66.5% girls), aged 10–14 years, recruited from secondary schools in Madrid, Spain. Depressive symptoms were assessed using the Children’s Depression Inventory-Short version (CDI-S), and physical–verbal aggression was measured with the Physical and Verbal Aggression Questionnaire (PVAQ). Repeated-measures analysis of variance (RM-ANOVA) was used to analyse changes in aggression across three approximately annual assessments (mean ages: 10.8, 11.8, and 12.8 years), with sex and depression status included as between subject factors.

**Results:**

Adolescents with depression consistently showed higher levels of physical–verbal aggression across all three assessment waves compared with those without depression. Aggressive behaviour followed a quadratic trajectory, increasing between ages 10.8 and 11.8 and decreasing at age 12.8, although elevated levels persisted in the presence of depression. Boys and girls exhibited similar developmental patterns; however, boys showed greater severity and persistence of aggression. The association between depression and physical–verbal aggression was independent of normative sex differences and emerged as early as 10.8 years of age, highlighting early adolescence as a key period for preventive screening and intervention.

**Conclusions:**

Adolescents with depression show a high and persistent physical–verbal aggression during early adolescence, with sex differences emerging primarily in severity rather than in the form of developmental trajectories. These findings underscore early adolescence as a key period for identifying adolescents at increased risk and highlight the value of longitudinal assessment for informing preventive and clinically relevant interventions targeting the co occurrence of depressive mood states and aggressive behaviour.

## Introduction

One of the less explored effects of depression, characterised in adolescence by severe mood disturbances such as sadness, hopelessness, low self-esteem, and anhedonia [1], is its potential association with increased aggression [2]. Accumulating evidence indicates that depressive symptoms are not exclusively internalising in nature but may also be associated with higher levels of extenalising behaviours, including both physical and verbal aggression [3]. This association may be explained by affective dysregulation processes, whereby increased irritability, reduced frustration tolerance, and difficulties in emotion regulation make adolescents more likely to externalise negative affect through aggressive responses rather than regulate it internally [4]. While studies have examined this relationship in childhood [5] and adolescence [6], there is a marked lack of longitudinal research focused specifically on early adolescence as a critical developmental transition [7].

Early adolescence, spanning ages 10 to 14 [8], is particularly critical as it marks the onset of sex differences in depression [9]. During this period, the prevalence of depressive symptoms becomes 1.5 to 2 times higher in girls compared to boys [10], and remain throughout the lifespan [11]. Despite this well-established divergence in depressive symptomatology, findings on whether depression-related aggression differs by sex during adolescence remain inconsistent. Some studies indicate that depression is associated with higher levels of aggression in girls [12, 13], while others suggest that it primarily affects boys [14, 15], and still others find no significant sex-based differences, with both groups equally affected by comorbidity [16, 17]. Taken together, these mixed findings suggest that sex differences in the depression–aggression link may reflect dynamic developmental and regulatory processes, rather than stable cross study patterns. According to Defoe [18], such discrepancies arise primarily due to overlooked moderation effects based on sex and the developmental phase [19]. Therefore, investigating how sex-related depression is associated with differences in physical and verbal aggressive behavior over time during a critical developmental stage such as early adolescence is essential to understanding its long-term impact.

Adolescent aggression is considered a serious public health problem [20]. Not only is it a precursor to the physical and mental health problems that victims will suffer, aggressive adolescents themselves are at greater risk of alcohol and drug abuse, violent crime, and suicide attempts [21]. Consequently, human aggression costs vast sums of money each year in healthcare, legal costs, and lost productivity, reaching $9.3 billion in US studies, while in Europe and the UK, the costs associated with adverse experiences are estimated at between €1.6 billion and €3.2 billion, respectively [22, 23]. Therefore, early identification of aggression is crucial [24], as evidence suggests that without aggression, both violence and crime escalation decrease significantly [25]. When aggression treatment and prevention prove ineffective, two serious consequences arise: an increased likelihood of persistence into adulthood [26] and a greater risk of dangerous behaviours [27]. Unfortunately, meta-analytic study reviewing therapeutic efficacy in adolescents indicate that interventions have no significant impact on recidivism among incarcerated adolescents [28]; and only a minor effect on aggression reduction in the general adolescent population [29]. Given the limited effectiveness of conventional treatments, Fairchild [30] suggests shifting the focus toward alternative approaches with empirical support that not only enhance intervention strategies but also contribute to prevention efforts. A key to directing objectives lies in the rates of mood disorders in detained violent youth, whose prevalence can reach up to 78% of these young people with problems in self-regulating their aggressive responses [31], highlighting depression as a particularly relevant but under-addressed target [32].

The relationship between depression and aggression among delinquent boys and girls shows a dangerous risk. Violent adolescents, delinquents, and criminals with depression exhibit significantly higher levels of aggression than their counterparts without depression [33]. Evidence suggests that by adulthood, the risk of committing a violent crime among people with depression, regardless of sex, is three times higher than in the general population [34]. Therefore, prevention efforts must therefore focus on adolescence, although the precise age at which intervention is most effective remains unclear due to conflicting findings in the literature. Moffitt [35] identified the ages of 14 to 16 as a critical period in which antisocial behaviour tends to increase, including child-to-parent violence [36]. However, studies in the general population indicate that children aged 11–12 are the most likely to engage in violent altercations with teachers [37, 38]. These inconsistencies show the need for longitudinal research to identify critical moments when depression shows effects that impact intraindividual change during adolescence.

In sum, despite substantial evidence linking depression and aggression, three important gaps remain: (a) a scarcity of longitudinal studies focused on early adolescence, (b) inconsistent findings regarding sex differences in depression-related aggression, and (c) limited consideration of both physical and verbal aggression within the same developmental framework. The present study addresses these gaps employing a three-wave longitudinal design in early adolescence (ages 10–14), examining the response of physical and verbal aggression associated with depression, and explicitly testing sex as a modulating factor in developmental trajectories. By doing so, this study provides novel evidence on when and for which sex, depression most strongly associates aggressive behaviour during this critical developmental window. Consequently, this longitudinal study pursued two primary objectives: (1) examine the longitudinal association between depression and physical and verbal aggression in early adolescence and (2) analyse the joint role of sex and depression in shaping the trajectories of physical and verbal aggression, identifying which sex is most strongly affected and how these patterns evolve throughout early adolescence.

## Materials and Methods

### Participants

This longitudinal study drew on data from adolescents recruited from ten public and private secondary schools in Madrid, Spain. During the study period from November 2007 to January 2010, a total of 525 participants were screened for eligibility. Based on the exclusion criteria, 274 adolescents were excluded due to lack of consent or incomplete protocols (defined as more than 30% missing responses). After applying these criteria, the final sample analysed for this longitudinal study consisted of 251 adolescents (66.5% female) who completed the three waves. At baseline (Time 1; T1), participants had a mean age of 10.86 years (standard deviation (SD) = 1.31), which increased to 11.86 years (SD = 1.31) in the second wave (Time 2; T2) and 12.86 years (SD = 1.31) in the third wave (Time 3; T3). Their ages ranged from 10 to 14 years, which aligns with the early adolescent developmental stage targeted by the study. According to the Hollingshead index [39], the majority of families belonged to the middle socioeconomic class (93.2%) and lived in two-parent households (91.2%). The baseline sociodemographic characteristics of the study sample (age group, sex, socioeconomic status, and family structure) are presented in Table 1.

**Table 1.** **Characteristics of the study sample**.

Characteristics		N	%
Gender of adolescent			
	Male	84	33.5%
	Female	167	66.5%
Age (years)			
	10–12	69	27.5%
	13–14	182	72.5%
Socioeconomic status			
	High	3	1.2%
	Middle	234	93.2%
	Low	14	5.6%
Single-parent family			
	Two-parent	229	91.2%
	Single-parent	22	8.8%

**Fig. 1.**
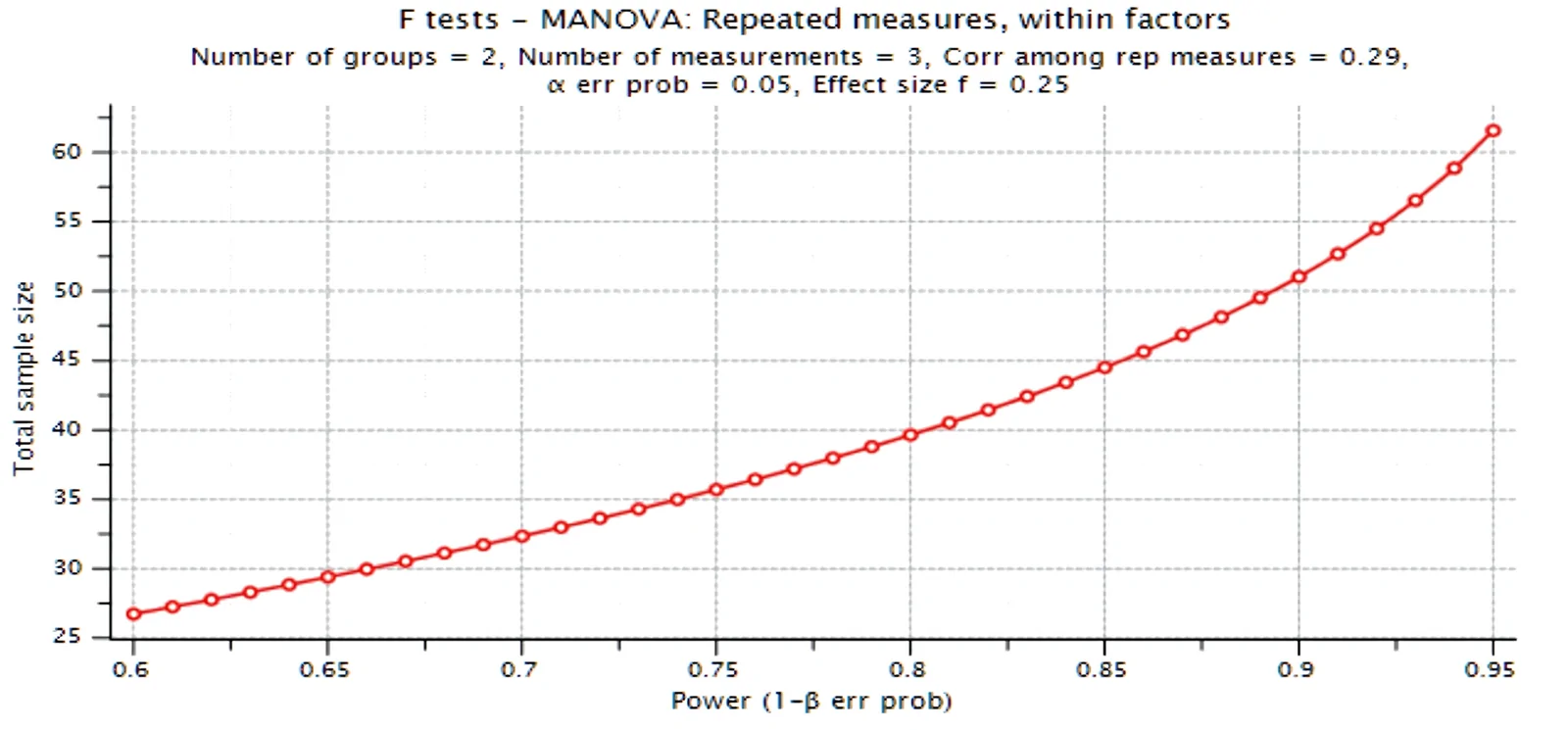
Result obtained by the G*Power program to estimate sample size. MANOVA, multivariate analysis of variance.

To calculate sample sizes, we used G*Power program (version 3.1.9.7; Heinrich-Heine-Universität Düsseldorf, Germany) [40]. Parameters were: effect size f = 0.25; α error problem = 0.05; Power (1-β err prob) = 0.95; number of groups = 2; number of measurements = 3; and correlation among repeated measures as Gordillo (2022) = 0.29. According to the program’s results, the sample size had to be equal to or greater than 62 adolescents (Fig. 1). Therefore, the final sample of 251 participants exceeded this requirement and ensured a balanced longitudinal design, minimizing the potential impact of partial attrition on the estimation of trajectories.

### Procedure

The sample was obtained using a multistage stratified random sampling procedure. In the first stage, schools were randomly selected from the official list of public and private schools provided by the Madrid Education Authority (cluster sampling). In the second stage, students within participating schools were selected using stratification by age and sex to ensure adequate representation of key demographic groups.

School principals were then contacted and provided with a summary of the study objectives and procedures, along with a formal request for participation. Of the 15 schools contacted, 10 agreed to participate and were included in the study. Although some institutional non response occurred, participating schools were comparable in type (public/private) and general characteristics to those initially selected, suggesting that the final sample remained broadly representative of the target population.

Within each participating school, a stratified sampling strategy based on sex and age was applied to ensure that the final sample reflected the demographic distribution of the student population. This approach enhanced the representativeness and generalisability of the findings. Once school participation was confirmed, principals or heads of studies facilitated contact with classroom tutors, who assisted in organizing the data collection process. In schools with Guidance Departments, educational psychologists also contributed to planning the assessment procedure. Written informed consent was obtained from parents or legal guardians through forms distributed by classroom tutors.

Assessments were conducted during regular school hours in group settings, with approximately 25 students per classroom, and were administered by trained psychologists following standardized instructions to ensure consistency across all settings. Before administration, participants received a brief explanation of the study and the evaluation instruments. The group-based administration and high participation rates within classrooms minimized the likelihood of systematic non-response bias at the participant level.

Exclusion criteria were applied as follows: Students with special educational needs were excluded from the analyses (although these students remained in the classroom and completed the questionnaires), as were those who did not provide parental consent, presented incomplete protocols (30% missing items), or did not complete all three assessment waves. The proportion of incomplete protocols was low (below 5% in all schools).

This research was conducted as part of the project “Trajectories of physical and verbal aggression in relation to the expression and regulation of anger from childhood to adolescence. A longitudinal study,” funded by the Spanish Ministry of Education and Science (State Secretariat for Universities and Research), grant number PIA 12007-11, and reviewed and approved by the Ethics Committee of the National University of Distance Education (UNED), approval reference SEJ2007-67522/PSIC.

### Instruments

Depression. Participants completed the Children’s Depression Inventory-Short version (CDI-S), derived from the original 27-item Children’s Depression Inventory developed by Kovacs [41] and adapted to the Spanish population by del Barrio *et al*. [42]. The CDI-S form is a self-report measure with 10 items rated on a 3-point scale (0–2), yielding a total score ranging from 0 to 20, with higher scores indicating greater depressive symptomatology. The items represent three levels of depressive intensity in the emotional state experienced during the past two weeks in negative self-esteem (e.g., “Nobody loves me”), anhedonia (e.g., “I’m always sad”) and hopelessness (e.g., “I’ll never do anything right”). Internal consistency (Cronbach’s α) obtained in this study was 0.73, 0.73, and 0.74 at T1, T2, and T3, respectively. The original cutoff point for participants aged 9 to 11 years is 7.04, and for those aged 12 to 15 years, it is set at a raw score of 8.24. For analytical purposes, depressive state (presence versus absence) was defined by a dimensional criterion, and participants who obtained a score higher than the sample mean plus one standard deviation on the CDI-S were classified as adolescents with the presence of depression.

Physical and verbal aggression was measured using the Physical and Verbal Aggression Questionnaire (PVAQ) [43]. Spanish adaptation by del Barrio *et al*. [44]. PVAQ consists of 20 items that measure different aggressive behaviours, both physical (e.g., “Kicking and punching”) and verbal (e.g., “Cursing”, “Threatening others”). Responses are rated on a 3-point scale (often, sometimes, never). Of the 20 items, 15 contribute to the total score, whereas 5 are control items and are not included in the scoring procedure. Consequently, total scores range from 15 to 45, with higher scores indicating greater physical and verbal aggression. Its application is individual or collective for children and adolescents aged 7 to 17 years. Internal consistency (Cronbach’s α) obtained in this study was 0.81, 0.83, and 0.80 (physical aggression) and 0.75, 0.78, and 0.74 (verbal aggression) in T1, T2, and T3, respectively.

### Statistical Analysis

Data were analyzed using IBM SPSS Statistics, version 26 (IBM Corp., Armonk, NY, USA). A repeated measures analysis of variance (RM-ANOVA) was conducted to analyze the longitudinal trajectory of physical and verbal aggression, allowing for the estimation of group mean comparisons across multiple time points. The three measurement occasions were approximately equally spaced, with assessments conducted at one-year intervals (mean ages: 10.8, 11.8, and 12.8 years), resulting in a balanced longitudinal design appropriate for the application of RM-ANOVA. Normality was evaluated using skewness, kurtosis, and visual inspection; although formal tests were significant, deviations were moderate and deemed acceptable given the robustness of RM-ANOVA and its within-subject structure, which reduces error variance. Sphericity was assessed using Mauchly’s test and, when violated, corrected using Greenhouse–Geisser estimates. No data transformations were applied. Potential outliers were screened via standardized scores and boxplot inspection, with no influential cases identified. Post hoc comparisons were performed using Bonferroni-adjusted significance levels. Analyses were conducted on complete cases only, ensuring a balanced repeated-measures design. All statistical tests were conducted using a significance level of α = 0.05, and all tests were two sided.

The between-subjects and within-subjects factors characteristic of RM-ANOVA that allow the longitudinal analysis of intraindividual change and interindividual differences in this study were the following (Table 2): age served as the within-subject factor, while sex and depression (presence/absence; scores above vs. below the mean plus one standard deviation) were included as between-subject factors, enabling group comparisons. The inclusion of sex and depression also allowed the examination of their interaction, ensuring that the effects of depression on aggression were estimated while accounting for normative sex differences in aggressive behaviour. Interactions between sex and depression were explicitly examined across time (i.e., age × sex, age × presence/absence depression, and age × sex × presence/absence depression), allowing us to determine whether the impact of depressive symptoms on aggression differed between boys and girls beyond baseline sex-related differences.

**Table 2.** **Design of the RM-ANOVA on physical and verbal aggression in early adolescence**.

Factor type	Variable	Levels / Categories
Within‑subject factor	Age (time)	T1 (10.8 years), T2 (11.8 years), T3 (12.8 years)
Between‑subject factor	Sex	Boys, girls
Between‑subject factor	Depression status	Absence, presence

Note: RM-ANOVA, repeated-measures analysis of variance; SD, standard deviation. Depression status was defined using a dimensional criterion (scores above vs. below the sample mean + 1 SD on the CDI-S). Age represents repeated assessments conducted at approximately one year intervals.

## Results

### Effects by Depression

**Fig. 2.**
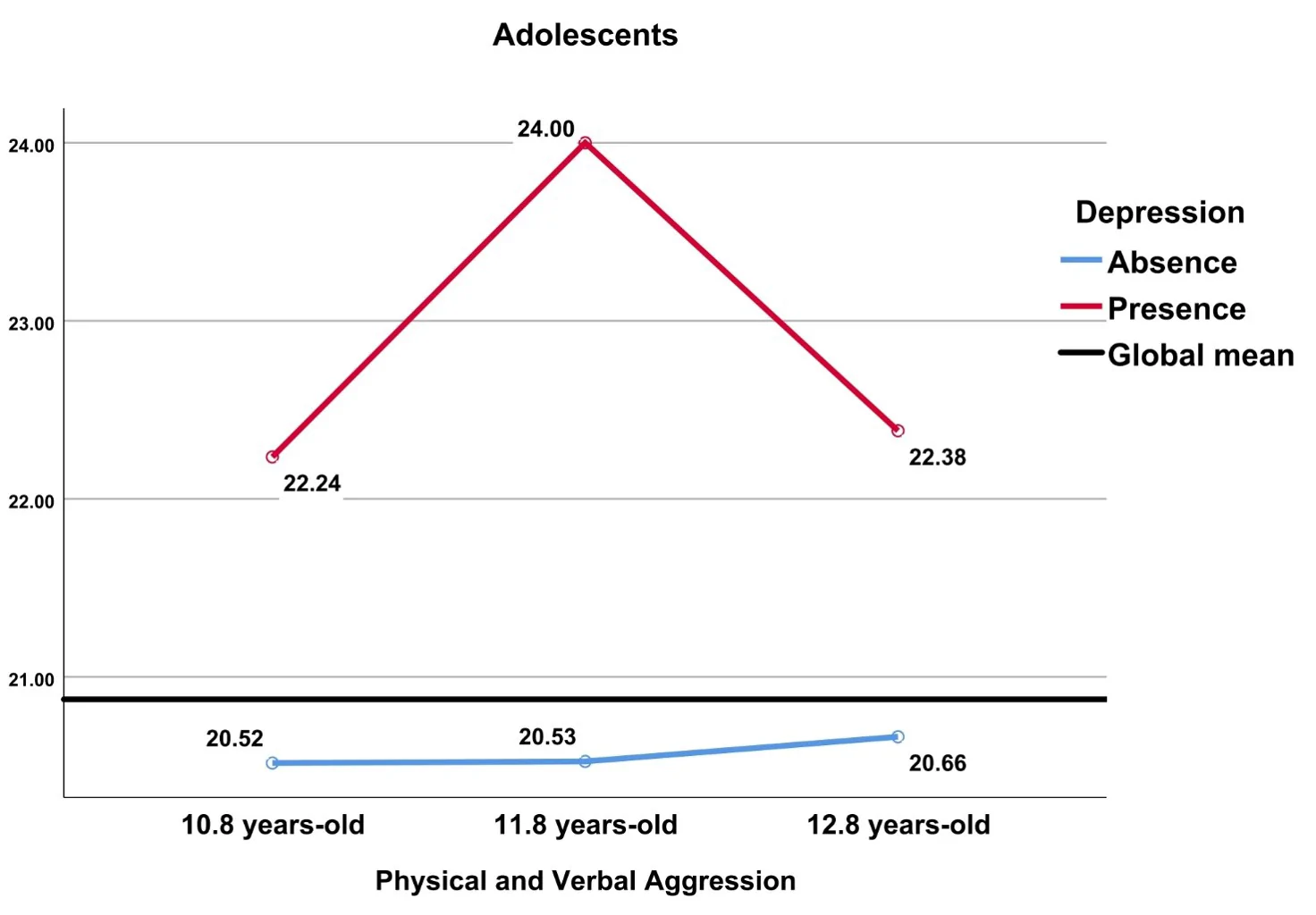
**Trend analysis of physical and verbal aggression in adolescents with and without depression in early adolescence**. The global mean represents the overall mean level of physical and verbal aggression across the full study sample included in the analysis.

According to the results of the within-subject effects tests (Table 3) and after verifying the sphericity assumption of Mauchly (W of Mauchly = 0.98; Chi-square = 3.13; *p* = 0.209), the data from this longitudinal study reveal a significant interaction between age and depression status (presence/absence) in responses to physical and verbal aggression among adolescents [(F(1, 249) = 8.20, *p* = 0.005, partial $\eta^{2}$ = 0.032)]. This finding indicates that physical and verbal aggression responses draw a quadratic trajectory change with a small to medium effect size, when depression is present during early adolescence. As illustrated in Fig. 2, adolescents with depression show, along a trajectory above the global average, an increase in physical and verbal aggression from 10.8 and 11.8 years, followed by a decrease from 11.8 and 12.8 years, returning to baseline levels.

**Table 3.** **Within-subject effects tests in physical and verbal aggression during early adolescence**.

Variables	Type III sum of squares	df	Mean square	F	*p*-value	Eta squared ($\eta^{2}$)
Age	45.10	2	22.55	2.33	0.099	0.009
Age*sex	9.13	2	4.56	0.47	0.625	0.002
Age*absence/presence depression	70.51	1	70.51	8.20	0.005	0.032
Age*sex*absence/presence depression	18.35	2	9.17	0.95	0.389	0.004

Note: df, degrees of freedom.

This longitudinal study revealed a consistent association between depression and physical and verbal aggression over time. As shown in Fig. 2, adolescents with depression score above global mean thresholds at the three time points of measurement, whereas their non-depressed peers remained below those thresholds throughout. The between-subjects effects tests (Table 4) indicated that 13.5% of adolescents with depression (N = 34) exhibited on average during the measured period of early adolescence (M = 22.87, SD = 0.56), significantly higher scores in physical and verbal aggression [(F(1, 249) = 14.54, *p* < 0.001, partial $\eta^{2}$ = 0.055] than those adolescents without depression (M = 20.57, SD = 0.22). In short, depressive symptoms were associated with consistently higher levels over time with a moderate effect size on aggressive physical and verbal responses of adolescents.

**Table 4.** **Between-subject effects and simple effects of depression and sex on physical and verbal aggression**.

Variable	Mean square	df	F	*p*-value.	Categories	n	Mean	SD	Prevalence	Eta squared ($\eta^{2}$)
Depression	482.04	1	14.54	<0.001	Presence	34	22.87	0.56	13.5%	0.055
					Absence	217	20.57	0.22		
Sex	189.96	1	6.05	0.015	Boys	84	22.77	0.52		0.024
					Girls	167	21.21	0.36		
Girls	78.72	1	8.72	0.004	Presence depression	23	22.20	0.63	13.8%	0.050
					Absence depression	144	20.21	0.26		
Boys	257.89	1	6.43	0.013	Presence depression	11	24.27	1.10	13.1%	0.073
					Absence depression	73	21.27	0.43		

Note: df, degrees of freedom; SD, standard deviation. Rows labelled “Girls” and “Boys” represent simple effects of depression within each sex.

### Effects by Sex

**Fig. 3.**
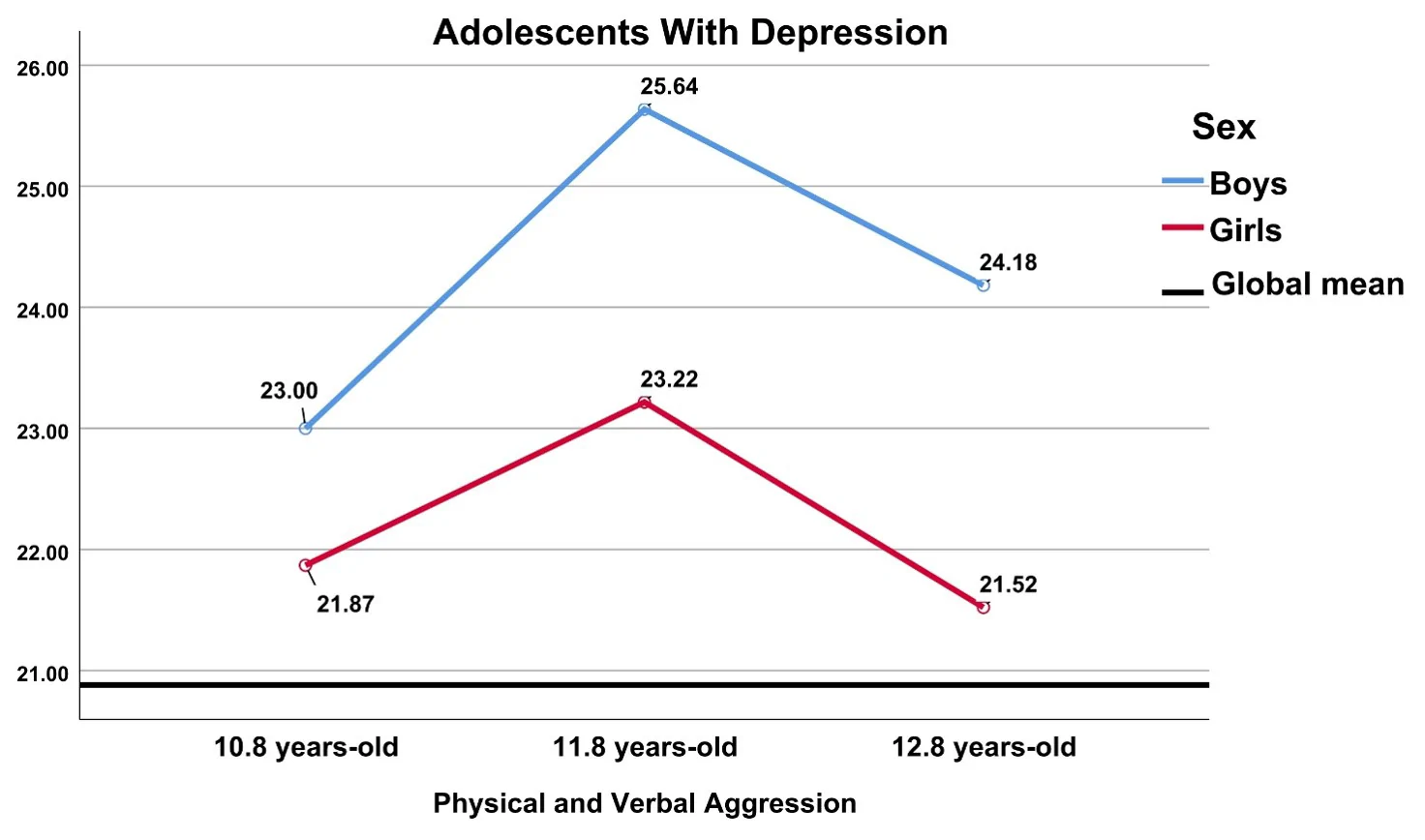
**Trend analysis by sex of physical and verbal aggression in adolescents with depression in early adolescence**. The global mean represents the overall mean level of physical and verbal aggression across the full study sample included in the analysis.

Regarding the sex variable, the within effects test analysis (Table 3) indicates that there isn’t significant interaction (F = 0.47, *p* = 0.625, partial $\eta^{2}$ = 0.002) and neither when analyzed in interaction with depression (F = 0.95, *p* = 0.389, partial $\eta^{2}$ = 0.004) This non-significant sex × depression interaction indicates that, although boys exhibited higher overall levels of aggression, the effect of depression on physical and verbal aggression was comparable across sexes after accounting for baseline sex differences. Depressed adolescents regardless of sex show the same developmental trajectory. As shown in Fig. 3, the physical–verbal aggression responses of depressed boys and girls are characterised by an increase between ages 10.8 and 11.8 followed by a decrease to age 12.8. Interestingly, although the results of longitudinal analysis show that adolescents and girls have a similar quadratic trajectory, they differ in severity and stability.

As shown in Fig. 3, adolescent boys maintained higher response rates than girls. In global mean, the between-subjects effects tests (Table 4) indicated that the scores for both groups differ significantly with a moderate effect size [F(1, 249) = 6.05, *p* = 0.015, partial $\eta^{2}$ = 0.024]. Specifically, adolescent boys (N = 84;) show significantly higher scores (M = 22.77, SD = 0.52) in physical and verbal aggression than girls (N = 167; M = 21.21, SD = 0.36). Furthermore, between 11.8 and 12.8 years of age, while the aggressive physical and verbal response in adolescent girls fell below the values at 10.8 years of age, the adolescent boys maintained scores above the values obtained in the first measurement. In conclusion, the results indicated that adolescents boys scored significantly higher and maintained these aggressive responses at higher levels throughout early adolescence.

### Effect of Depression in Girls

**Fig. 4.**
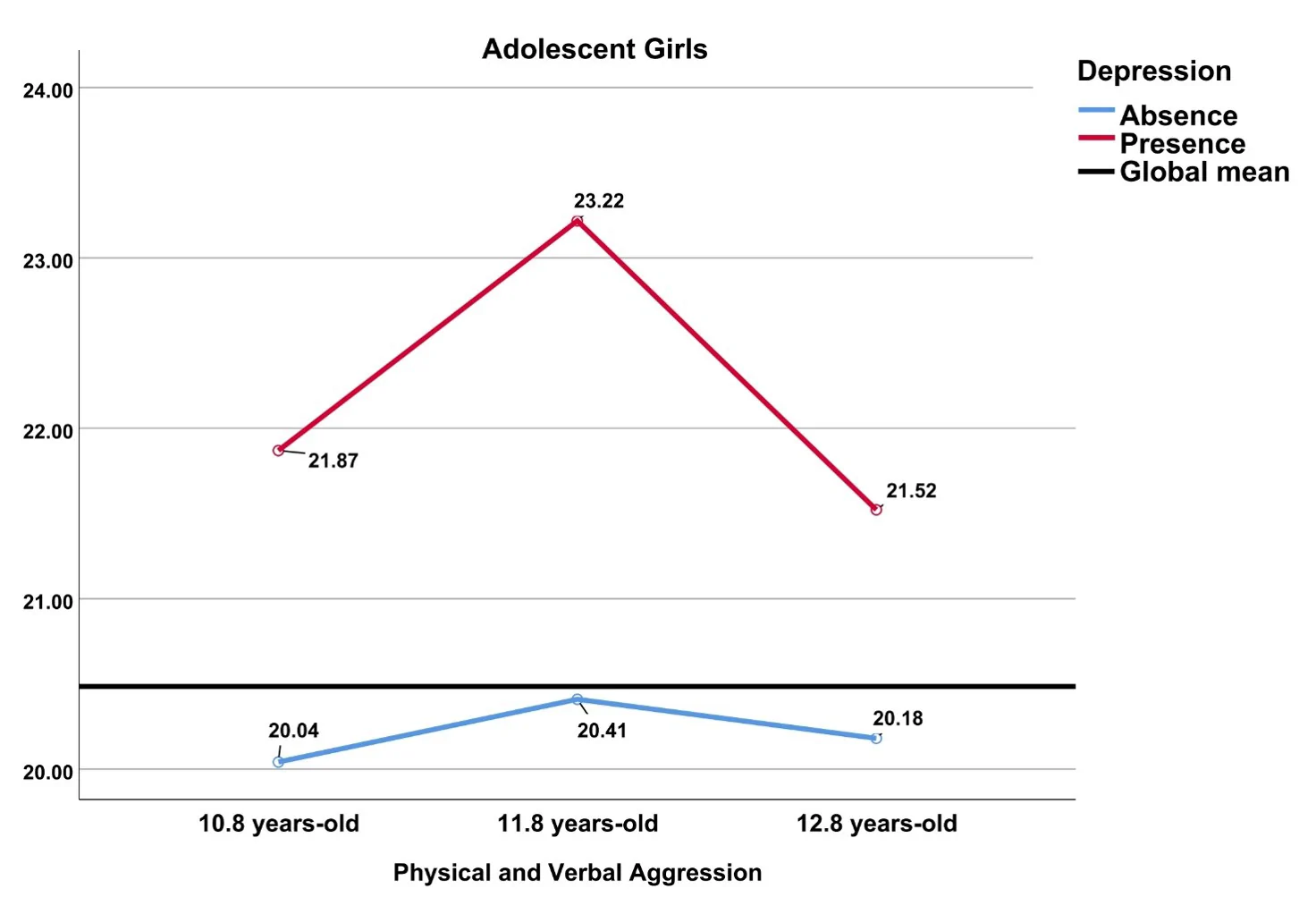
**Trend analysis of physical and verbal aggression in adolescent girls with and without depression in early adolescence**. The global mean represents the overall mean level of physical and verbal aggression across the full study sample included in the analysis.

The results indicated significant differences between adolescent girls with and without depression. Specifically, the results of the between-subjects effects test (Table 4) indicated the presence of depressive symptoms was significantly associated with higher levels of physical and verbal aggression [F(1, 165) = 8.72, *p* = 0.004, partial $\eta^{2}$ = 0.050; prevalence = 13.8%]. The depressed girls adolescent (n = 23) exhibited higher scores (M = 22.20, SD = 0.63) than their non-depressed counterparts (n = 144; M = 20.21, SD = 0.26). Effect size suggests a small-to-moderate magnitude; however, as shown in Fig. 4, adolescent girls with depression consistently scored above global mean throughout the entire developmental evaluation period (ages 10.8, 11.8, and 12.8). Another noteworthy result illustrated in Fig. 4, indicates that depression exacerbates the severity of the aggression pattern observed among girls without depression.

### Effect of Depression in Boys

**Fig. 5.**
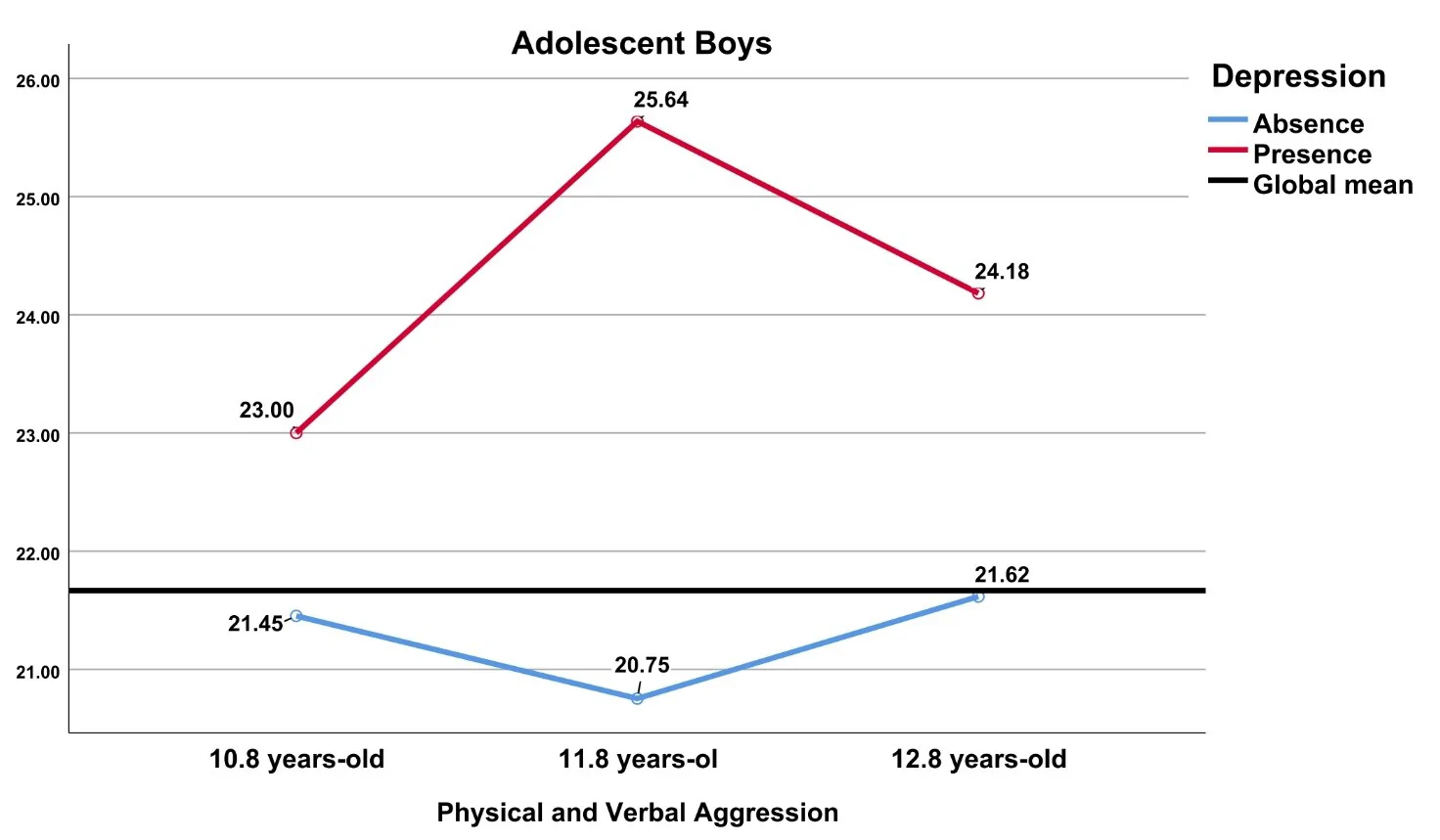
**Trend analysis of physical and verbal aggression in adolescent boys with and without depression in early adolescence**. The global mean represents the overall mean level of physical–verbal aggression across the full study sample included in the analysis.

Similarly to the results found in adolescent girls, the between-subjects effects test (Table 4) show boys with depressive symptoms exhibited significantly higher levels of physical and verbal aggression than those without depression [F(1, 82) = 6.43, *p* = 0.013, partial $\eta^{2}$ = 0.073; prevalence = 13.1%]. The mean score of the depressed group (n = 11) was higher (M = 24.27, SD = 1.10) than that of the non-depressed group (n = 73; M = 21.27, SD = 0.43). This effect size (partial $\eta^{2}$ = 0.073) is the largest observed in the analyses, indicating that depression was associated with higher levels of aggression in boys compared to girls. One finding is particularly noteworthy when compared to girls. As illustrated in Fig. 5, adolescent boys with depression reverse the response pattern of those without depression, which contrasts with the increased trajectory already observed in girls without depression (Fig. 4). This result indicates that, at 10.8 years of age, boys with depression show an increase in aggressive behavior with values above the global average, while, in the absence of depression, aggressive behavior at this age shows a tendency to decrease.

## Discussion

This study provides longitudinal evidence that depressive symptoms are associated with elevated and persistent patterns of physical and verbal aggression across early adolescence. Specifically, adolescents with depression showed consistently higher aggression levels across all three measurement waves, following a quadratic developmental trajectory characterised by an increase between ages 10.8 and 11.8 and a partial decline at age 12.8. These findings support and extend previous research by clarifying the timing, persistence, and sex-specific expression of depression-related aggression during early adolescence.

In line with the first objective of the study, the findings showed significant differences in aggression levels between adolescents with and without depression over time, starting from the first measurement point (10.8 years). Furthermore, once aggression emerges in the depressed group, it remains above the global mean during the first three years of early adolescence. The fact that the data from this study show that adolescents without depression are associated with below average responses suggests that depression could be a potential treatment target for these young people [45], especially considering the low efficacy of therapeutic programs focused on aggression [26]. Furthermore, the early age detected (10.8 years) suggests difficulties in emotional regulation mechanisms. Two interesting theoretical frameworks whose epistemological core is based on emotional dysregulation, defined in relation to difficulties in modulating the intensity of emotions, inhibiting impulsive responses, and recovering after a negative emotional state, are the Sense of Coherence (SOC) and the Sense of Agency (SoA). Both frameworks, the SOC focus on low self-esteem and a diminished sense of meaning [46, 47], alongside the SoA, which focuses on the subjective experience of volitional control to mitigate emotional detachment between anger and its typically aggressive consequences [48, 49], emerge as promising approaches to address the effects of depression in adolescents with high levels of physical and verbal aggression [50, 51]. In short, this study adds to previous research by supporting the relevance of incorporating preventive learning about affective mechanisms of depression into clinical guidelines, with the aim of helping adolescents understand how sadness maybe associated with aggressive behaviours and what resources they have to deal with it.

Longitudinal analysis of the association between depression and physical and verbal aggression showed a distinctive quadratic trajectory of physical and verbal aggression, exceeding global mean thresholds. Specifically, aggression levels increased between the ages of 10.8 and 11.8 years, followed by a decrease at age 12.8 years. This pattern is consistent with previous evidence that focuses on assessing depression as a dynamic variable for externalizing behaviors rather than a static variable. These findings highlight the need for longitudinal assessment frameworks capable of capturing critical inflection points in symptom trajectories–patterns that might otherwise be misinterpreted within cross-sectional models [52]. Therefore, the persistence of high levels of aggression in depressed adolescents underscores the need for diagnostic approaches that address the chronicity of symptoms beyond the acute management of overt aggressive behaviors. In summary, the longitudinal trend analyses in this study provide valuable insights into developmental trajectories, reinforcing the crucial role of longitudinal methodologies in adolescent research [53]. Notably, the data from this study show that while aggression levels decrease between ages 11.8 and 12.8, they remain at the same level as responses reported at age 10.8, marking a significantly higher baseline than adolescents without depression. This further supports the importance of continuous follow-up, since cross-sectional assessments may underestimate the continuity of this comorbidity rather than accurately capturing its severity and longitudinal dynamism.

Regarding the second objective of the study, the findings indicate that, although boys and girls exhibited similar developmental trajectories of physical and verbal aggression across early adolescence—characterised by a comparable quadratic pattern of increase followed by a decline—important differences emerged in terms of severity and persistence. Specifically, boys displayed higher overall levels of aggression and a less pronounced decline over time compared to girls. This distinction between trajectory shape (i.e., the pattern of change over time) and trajectory level (i.e., the overall magnitude of aggression) helps clarify that similar developmental forms can coexist with meaningful sex differences in intensity and chronicity. The similarity in trajectory shape is consistent with previous longitudinal research reporting comparable developmental patterns of aggressive behaviour in boys and girls [54]. However, the observed differences in severity align with findings from community samples, where depressed adolescent boys tend to show higher levels of physical aggression [55]. In the present study, while girls showed a marked decline between ages 11.8 and 12.8—returning to baseline levels—boys maintained elevated aggression above initial levels, suggesting greater stability of aggressive responses when depressive symptoms are present. These findings concerning the chronicity of physical and verbal aggression during early adolescence, particularly among boys, are consistent with Farrington’s perspective that delinquent behavior does not appear abruptly but rather evolves along an early and progressive aggressive–antisocial trajectory [56]. They also fit within his developmental crime prevention framework, which emphasizes the importance of identifying “who is at risk?” as a central principle for informing prevention efforts, highlights the importance of early detection of depression in aggressive youth [57].

This study has some limitations and strengths. Firstly, our data were based on self-reports, so future research should incorporate data from multiple sources and increase validity [58]. In addition, although descriptive information on socioeconomic status and family structure was collected, these variables, together with other potentially relevant factors such as parenting practices, exposure to bullying, or broader psychosocial adversities, were not included as covariates in the analyses. These factors may influence both depressive symptoms and aggressive behaviour and, therefore, their omission may limit the precision of the estimated associations. However, the longitudinal repeated-measures design partly mitigates this limitation by allowing the examination of intra-individual change over time, reducing the influence of stable between-subject confounders. Secondly, given that the frequency of antisocial and aggressive behaviours often increases during mid-adolescence (approximately ages 14–16), future longitudinal studies should extend follow-up into later adolescence to capture potential changes in trajectory and severity across this critical period. Finally, for theoretical reasons, the findings of the present study should be limited to general symptoms of depression and aggression (physical and verbal) using a dimensional approach. Among the strengths that this study presents, is the inclusion of verbal aggression in the explanatory model. Surprisingly, verbal aggression is often excluded from the general measure of aggression, when this type of aggression in the general and clinical adolescent population is the most frequent in both everyday situations and school violence [59, 60].

## Conclusions

In this study, the risk of elevated physical and verbal aggression was found to be unevenly distributed in early adolescence, with depressive mood states marking a particularly vulnerable developmental profile. Adolescents with depression consistently showed higher levels of aggression over time, and this pattern was especially pronounced among boys, who exhibited greater severity and persistence despite following a developmental trajectory similar in shape to that of girls. These findings indicate that the vulnerability to aggressive behaviour in early adolescence is not uniform but rather reflects the interaction between mood state, sex, and developmental timing. Therefore, identifying at-risk adolescents, particularly those with early depressive moods and persistent aggressive responses, can help clinicians, educators, and other policymakers refine early detection practices and prioritize prevention efforts based on sex.

## Availability of Data and Materials

Gordillo, Rodolfo (2026), “Depression as a Longitudinal Modulator of Physical–Verbal Aggression in Early Adolescence: Trajectories and Sex Comparisons”, Mendeley Data, V3, doi: 10.17632/8yxrztg233.3, under licence: CC BY-NC-ND 4.0. This license enables reusers to copy and distribute the material in any medium or format in unadapted form only, for noncommercial purposes only, and only so long as attribution is given to the creator.
